# Acknowledgement of January – September 2013 peer reviewers of *Journal of Medical Radiation Sciences*

**DOI:** 10.1002/jmrs.31

**Published:** 2013-12-10

Publishing a quality peer-reviewed journal relies on the contribution of many peer reviewers. The members of the Editorial Review Board – especially the editors – acknowledge the −contribution of the following colleagues who worked behind the scenes of *Journal of Medical −Radiation Sciences* in January – September 2013. Thank you for your passion towards improving and maintaining the quality of publications in *Journal of Medical Radiation Sciences*.

Mohd Hanafi Ali

Berry Allen

Elizabeth Bailey

Luke Barclay

Charlotte Beardmore

Robin Bell

Lisa Booth

Scott Bowman

Julie Burbery

David Christie

Andrew Cousins

Jenny Cox

Agnella Craig

Rob Davidson

Karen Dobeli

Edgar Estoesta

Craig Everitt

Mark Farnworth

Rhys Fitzgerald

Diana Gentilcore

Shaun Grady

Kareen Grimshaw

Hazel Harries-Jones

Jill Harris

Robin Hart

Suzanne Henwood

Peter Hogg

Daphne James

Paul Kane

Peter Kench

Tracy Kirkbride

Karen Knapp

Peter Lavery

Joerg Lehmann

Sarah Lewis

Andrew Mallitt

Kristie Matthews

Clare Monk

Laura Mullaney

Martin Necas

Hazel Neser

Curtis Ng

Rebecca Owen

Nick Phillips

Suresh Rana

Andrew Renner

Taryn Robinson

Jenny Sim

Tony Smith

Pauline Soh

Zhonghua Sun

Jonathan Sykes

Kerry Thoirs

Hannah Thompson

Christine Vanderley

Gillian Whalley

Kate Wilkinson

Stephanie Wrightson

Bosco Yu

